# A longitudinal magnetic resonance imaging study of neurodegenerative and small vessel disease, and clinical cognitive trajectories in non demented patients with transient ischemic attack: the PREVENT study

**DOI:** 10.1186/s12877-018-0858-4

**Published:** 2018-07-16

**Authors:** Sana Tariq, Christopher D. d’Esterre, Tolulope T. Sajobi, Eric E. Smith, Richard Stewart Longman, Richard Frayne, Shelagh B. Coutts, Nils D. Forkert, Philip A. Barber

**Affiliations:** 1 Calgary Stroke Program, Department of Clinical Neurosciences, Foothills Medical Centre, 1403 - 29 Street NW, Calgary, AB Canada; 2 Seaman Family MR Center, Foothills Medical Centre, 1403 29th Street NW, Calgary, AB Canada; 3 Department of Community Health Sciences & O’Brien Institute for Public Health, University of Calgary, 3280 Hospital Drive NW, Calgary, AB Canada; 4 Hotchkiss Brain Institute, Foothills Medical Center, Room 1A10 Health Research Innovation Center, 3330 Hospital Drive NW, Calgary, AB T2N 4N1 Canada; 5 Department of Radiology and Clinical Neurosciences, Cumming School of Medicine, University of Calgary, Calgary, AB Canada

**Keywords:** Magnetic resonance imaging, Neurodegeneration, Alzheimer’s disease, Vascular dementia, Small vessel disease, Cognition, Aging

## Abstract

**Background:**

Late-life cognitive decline, caused by progressive neuronal loss leading to brain atrophy years before symptoms are detected, is expected to double in Canada over the next two decades. Cognitive impairment in late life is attributed to vascular and lifestyle related risk factors in mid-life in a substantial proportion of cases (50%), thereby providing an opportunity for effective prevention of cognitive decline if incipient disease is detected earlier. Patients presenting with transient ischemic attack (TIA) commonly display some degree of cognitive impairment and are at a 4-fold increased risk of dementia. In the Predementia Neuroimaging of Transient Ischemic Attack (PREVENT) study, we will address what disease processes (i.e., Alzheimer’s vs. vascular disease) lead to neurodegeneration, brain atrophy, and cognitive decline, and whether imaging measurements of brain iron accumulation using quantitative susceptibility mapping predicts subsequent brain atrophy and cognitive decline.

**Methods:**

A total of 440 subjects will be recruited for this study with 220 healthy subjects and 220 TIA patients. Early Alzheimer’s pathology will be determined by cerebrospinal fluid samples (including tau, a marker of neuronal injury, and amyloid β_1–42_) and by MR measurements of iron accumulation, a marker for Alzheimer’s-related neurodegeneration. Small vessel disease will be identified by changes in white matter lesion volume. Predictors of advanced rates of cerebral and hippocampal atrophy at 1 and 3 years will include in vivo Alzheimer’s disease pathology markers, and MRI measurements of brain iron accumulation and small vessel disease. Clinical and cognitive function will be assessed annually post-baseline for a period of 5-years using a clinical questionnaire and a battery of neuropsychological tests, respectively.

**Discussion:**

The PREVENT study expects to demonstrate that TIA patients have increased early progressive rates of cerebral brain atrophy after TIA, before cognitive decline can be clinically detected. By developing and optimizing high-level machine learning models based on clinical data, image-based (quantitative susceptibility mapping, regional brain, and white matter lesion volumes) features, and cerebrospinal fluid biomarkers, PREVENT will provide a timely opportunity to identify individuals at greatest risk of late-life cognitive decline early in the course of disease, supporting future therapeutic strategies for the promotion of healthy aging.

## Background

Cognitive impairment late in life is commonly caused by progressive neuronal loss related to two dominant diseases, Alzheimer’s disease (AD) and small vessel cerebrovascular disease (i.e. vascular dementia; VaD), both causing brain atrophy years before clinical symptoms are detected. As dementia is attributed to vascular and life-style related risk factors in mid-life in a substantial proportion of patients (50%), there is an opportunity to modify these factors if incipient disease is detected earlier [1]. Transient ischemic attack (TIA; incidence 0.37–1.1 per 1000) [2, 3], is associated with a 4-fold increase in dementia over the general population [4–6]. Therefore, TIA patients are a potential target for early modification of incipient disease processes that precede cognitive impairment guided by biologically relevant markers such as amyloid beta 1–42/ tau, whole brain and sub-structure atrophy, small vessel disease markers, and increased free iron. The Predementia Neuroimaging of Transient Ischemic Attack (PREVENT) study will focus on quantifying these biomarkers using advanced serial magnetic resonance imaging (MRI), neuropsychological assessments, APOE genotyping, and longitudinal clinical data. Specifically, PREVENT will examine what combination of pathological processes and risk factors lead to cognitive decline after TIA.

## Methods

PREVENT is a prospective, single-center longitudinal study. To achieve a 30% group effect size on annualized rates of cerebral brain atrophy, *N* = 220 TIA patients and *N* = 220 healthy controls will receive clinical, neuropsychological, fluid biomarker, and MR assessments at pre-specified time points. The frequency and duration of events is summarized in Table 1.

**Table 1:** Schedule of Events

Visit Name	Baseline	Month 12	Year 3	Annual Review	Interim Review
Inclusion and Exclusion Criteria	•				
Medical History, Physical Exam	•	•	•	•	
Vital (BP, Heart rate etc.,) Signs	•	•	•	•	
DNA Sample Collection for APOE	•				
Neuropsychological assessment		•	•	•	
Activities of Daily Living (FAQ)		•	•	•	
Plasma and Serum Biomarker Collection	•	•	•	•	
Concomitant Medications	•	•	•	•	•
Adverse Events	•	•	•	•	•
3 T MRI Imaging (100%)	•	•	•		
CSF Collection by Lumbar Puncture	•				

### Patient and control subject populations

The inclusion criteria for the TIA patient cohort are: 1) First documented TIA as defined by the following: resolved symptoms attributable to the anterior circulation (motor, speech, monocular visual loss), or posterior circulation (two or more of: ataxia, diplopia, vertigo, hemi-weakness, hemianopia) [2]; 2) no dementia as defined by National Institute of Aging-Alzheimer Association Criteria [7]; 3) clinical brain MR within 10 days of TIA symptoms to determine presence of diffusion-weighted image (DWI) lesion; 4) an age range between 45 and 75 years; and 5) fluency in English language.

Patient exclusion criteria are as follows: 1) persistence of stroke symptoms > 24 h [2]; 2) dementia as defined by the National Institute of Aging-Alzheimer Association Criteria [7]; 3) other central nervous system disease (e.g. MS), alcoholism, substance abuse, sedatives, antipsychotic medications and history of psychiatric illness; 4) contraindication to MR imaging, and other comorbidities that could significantly interfere with cognitive performance and follow-up (e.g., recent coronary artery bypass surgery (within 6 months), severe ischemic heart disease, heart failure, poorly controlled diabetes, renal or liver failure etc.).

Healthy controls will include volunteers from the community, and spouses of patients. Exclusion to participation includes prior stroke or TIA, dementia, co-morbid disease etc. as per patient exclusion criteria.

### Data collection

Baseline evaluation (time point: Year 0; Y0) will include clinical review, fasting cholesterol, glucose, and renal function tests. Treatments for hypertension, diabetes, hyperlipidemia, anti-thrombotic agents, and other medications that could influence cognition such as sedatives, anxiolytics, or psychotropic medications will be determined. Apolipoprotein E (APOE) genotyping will be performed; the APOE ε4 allele is a risk factor for AD and VaD [8]. Obstructive sleep apnea may affect cognition and will be screened [9]. Blood pressure (BP) measurement will include two sitting BP measurements taken 5 minutes apart. At enrollment, patients and healthy controls will have home BP monitoring training and will perform three readings per day (waking, mid-morning, and evening) with a Bluetooth enabled, telemetric system (A&D Model UA 767BT) during the first month of recruitment [10]. All TIA patients will be managed according to current stroke prevention guidelines [11]. Cerebrospinal fluid (CSF) for AD biomarkers, amyloid β_1–42_ and tau, will be collected according to a standardized procedure [12, 13]. The PREVENT study consent informs the participant that all results from biological tests (i.e. APOE) will not be disclosed to the patient or to the patient’s general practitioner. Participants will be approached about brain donation.

### Imaging acquisition

At study entry **(**Y0**)** patients will have MR scan as a standard of clinical care within 1–2 days of physician assessment, and then repeated at 1 (Y1) and 3 years (Y3). Healthy controls will have MR scan at the same time points (Y0, Y1, Y3). DWI will be performed to detect evidence of diffusion lesion after TIA (up to 30%) [14]. The pertinent biological imaging targets of the standard MR imaging acquisition are summarized in Table 2. The absolute brain and hippocampal volume change between Y0, Y1, and Y3 will be calculated from T1-weighted images with SIENA [15] and FIRST [16, 17] part of FSL software package [18, 19]. At baseline and follow up, FLAIR images will be registered to high-resolution T1 images using a rigid transformation and optimization of the mutual information. Quantitative Susceptibility Mapping (QSM) measurements of regional brain iron accumulation will be acquired using methodology developed and published by the Calgary Image Processing and Analysis Center (CIPAC) [20].

**Table 2:** PREVENT imaging comprehensive dementia protocol < 20 min of scan time

Imaging Sequence	Mechanistic Target	Endpoint	Geometric Parameters (2D/3D, FOV)	Acquisition Parameters (TE/TR/flip)
3-plane localizer			2D, 24 cm, 8 mm/ 8 mm, 33, 256 × 128	1.3 ms/ 4.8 ms/ 30°
High Resolution T1-weighted	Atrophy	Primary and secondary outcome	3D, 24 cm^2^ 2.0 mm/ 0 mm, 70, 256 × 256	3 ms/ 7 ms/ 8° (minTE, minTR)
3D T2-weighted FLAIR	WML volume	Predictor of primary outcome	3D, 24 cm, 1 mm/ 0 mm, 38, 256 × 256	140 ms/ 9000 ms/ 90°
Axial 3D QSM	Neurodegeneration Brain iron content	Predictor of primary outcome	3D, 24 cm, 2 mm/0 mm, 192 × 192	29.5 ms/3 ms/8°
3D Proton Density	WML volume		3D SPGR, 1 mm/ 0 mm, 256 × 256	
Diffusion tensor DWI	Microstructure Acute cytotoxic ischemia		2D, 24 cm, 3.5 mm/ 0 mm, 38, 144 × 144 matrix	70 ms - 80 ms/ 13,000 ms/ 90°
Axial T2 star	Microhemorrhage		2D, 24 cm, 3.5 mm/ 0 mm, 38, 256 × 224	140 ms/ 9000 ms/ 90°7

### Neuropsychological assessment

Performed at baseline (within 10 days of onset in TIA patient) and then annually by a trained research assistant (Table 3). The cognitive tests are designed to measure change over time, have been selected to minimize floor and ceiling effects, can distinguish normal aging from prodromal dementia, and will be rotated to reduce practice effects. These tests are consistent with harmonization standards for vascular cognitive impairment (VCI) [21].

**Table 3:** PREVENT Neuropsychological Battery

Domain	Test	Description
Attention	Trail Making A [52]	Measure of attention, speed and mental flexibility through connecting numbers from 1 to 25 in ascending order.
Processing Speed	Digit Symbol Coding Test (WAIS-IV) [53]	Assessment consisting of 9 digit-symbol pairs followed by a list of digits. Subject instructed to fill in the corresponding symbol within 120 s.
Verbal Memory	WHO/UCLA’s Auditory Verbal Learning Test [54]	Measure of verbal learning and memory. Subject asked to repeat a list of words (List A) for 5 consecutive trials. An interference trial is introduced (List B) followed by immediate recall and a 25-min delayed recall.
Visual Memory	Brief Visuospatial Memory Test – Revised (BVMT) [55]	Measure of visual learning and memory. Target figures presented to subject for 10 s for 3 consecutive drawing trials. After a 25-min delay subject is asked to redraw target figures. Recognition trial is almost administered using 6 novel and 6 target figures.
Executive Function	Stroop Color Word Test [56]	Measure of executive function to assess cognitive control. Subjects instructed to read colors out loud while ignoring the printed words in 45-s.
	Trail Making B [52]	Measure of mental flexibility. Subject asked to connect alternating numbers (1–13) and letters (A-L).
	Clox-1: Executive Clock Drawing [57]	Measure of visual spatial construction and executive abilities; also screens for dementia. The subject is instructed to draw a clock with hands and numbers and a specific time.
Working Memory	Digit Span Forward [58]	Test of working memory. Subject is asked to repeat back a list of numbers verbatim as they increase in difficulty.
	Digit Span Backwards [58]	Test of working memory. Subject is asked to repeat the list of words in backwards order as they increase in difficulty.
Language	Boston Naming Test (Short form) [59]	Assessment of visual naming ability. Subject is asked to name 15 objects after being presented with an illustration.
	Complex Ideation Material [60]	Assessment of error detection that may be attributed to dementia. Subject is asked yes/no questions about areas that are considered general knowledge. Subject is then read two stories and asked yes/no questions post-comprehension.
Premorbid Intelligence	North American Adult Reading Test (NAART) [61]	Measure of premorbid intelligence. The subject is asked to read a list of 61 words out loud.
Mood	Center for Epidemiological Studies - Depression Scale (CES-D) [62]	Self-reported questionnaire. The subject is asked to fill out a questionnaire that pertains to their mood and well-being.
Cognitive Screening Tests	Montreal Cognitive Assessment (MoCA) [63]	Assessment of multiple cognitive domains; sensitive to mild cognitive impairment and dementia.
	Addenbrooke’s Cognitive Assessment – Revised (ACE-R) [64]	Assessment of multiple cognitive domains; sensitive to MCI and dementia. The test has a built in mini mental state examination (MMSE).

### Power analyses

The PREVENT study will recruit 220 TIA patients to participate in this study and compare them to 220 controls, which is inclusive of 20% attrition rate in the whole sample, informed by our preliminary data and by other well documented longitudinal studies [22–24]. The study is powered based on multiple linear regressions of the association between the primary outcome of interest, first year cerebral atrophy rate (R1), and group effect, after adjusting for other socio-demographic, clinical, and vascular risk factors, and other potential confounders. With a sample size of 440, the study will have at least 85% power to detect a 30% group effect size (i.e. TIA versus control) following baseline measurement and follow up measurement taken at Y1, informed by similar rates of whole brain atrophy in published reports of prodromal dementia [25, 26], and our pilot data [27, 28]. This sample size will provide at least 90% power to detect a standardized difference of 0.35 between TIA and control groups for change over time on the WHO/UCLA AVLT (verbal memory) and Trails A and B tests (processing speed/executive function).

### Statistical analyses

Statistical analysis will include descriptive statistics to compare the mean, median, standard deviations, and frequency distribution s of all variables.

A mixed repeated measure regression model will be used to model the association between overall cognitive domain specific and composite z-score variable measures at baseline, 1 year, and 3 years and patient group (TIA versus healthy control), after adjusting for demographic characteristics, baseline cognitive function, vascular risk factors mentioned before, in vivo biomarkers, and patient interactions as fixed effects covariates. The size of the standardized regression coefficients associated with each predictor will be used to quantify its relative importance in predicting rate of cognitive decline in patients versus controls. A random intercept will be included to account for subject-specific variation in cognitive scores. The 95% confidence intervals and the corresponding *p*-values for adjusted group effect on overall cognitive outcomes will be estimated.

Finally, we will develop novel prediction models based on machine learning and generalized estimating equation models to identify individual TIA patients with a high likelihood to develop cognitive decline. The basic idea of this is to predict occurrence of cognitive decline using prediction models developed and trained on the longitudinal data with known clinical outcome (cognitive decline yes / no), which can then be used to predict cognitive impairment in new patients based on the same features used for training of the high-level machine learning model. More precisely, we will develop prediction models based on random forest [29], support vector machines [30], deep neural networks [31], and quadratic inference function classifiers for predicting the occurrence of dementia using available baseline information such as cognitive test results, blood and CSF parameters, as well as quantitative image-based biomarkers such as regional QSM values, volumetric brain and hippocampal values, and white matter lesion (WML) load. The evaluation of the prediction models based on the TIA and control cohorts will be conducted using well-established cross validation techniques. This means that we will, for example, exclude one patient with known dementia outcome from the training set. After this, the prediction model trained based on the remaining patients can be used to predict dementia outcome for the one patient not part of the training set and the predicted outcome can be compared to the real outcome. By iteratively repeating this so-called leave-one-out cross validation for all patients available, the accuracy of the prediction model can be determined and used to identify the optimal high-level machine learning method for this purpose.

## Discussion

In light of social and economic burdens, early identification of high-risk patients for dementia is the most important step in prevention and postponement [32, 33]. With no cure and recent clinical trial failures, it is becoming increasingly important that trials must be redesigned with a focus on high-risk populations and standardized biomarkers, which include clinical, demographic, imaging and neuropsychological considerations. The PREVENT study proposes the early identification of people at risk for late-life cognitive decline as the single most important approach for trial designs. To date, studies have established a link between vascular risk and stroke, and late life cognitive impairment but overlooked fundamental challenges such as the efficacy of vascular reduction treatment (antihypertensive treatments [34–38], diabetes management, and cholesterol lowering therapy [39]) on slowing cognitive decline, thus leading to inconclusive results. Special consideration needs to be given to identifying patients at the highest risk of dementia (i.e. TIA patients) since these patients are most likely to benefit from earlier intervention [40] before symptoms manifest, and from therapies that focus on vascular risk reduction [34]. Intervention trials such as the Finnish Geriatric Interventions to Prevent Cognitive Impairment (FINGER) study and the Prevention of Dementia by Intensive Vascular care (preDIVA) trial, show that focusing on diet, exercise, cognitive training, and vascular risk management as a multi-domain treatment approach improves or maintains cognitive function in at risk elderly when compared to healthy controls [24, 41]. The potential mechanisms of disease progression (AD, vascular disease or both), response of these multi-domain therapeutic interventions, and their long-term benefits remain unknown [42]. However, epidemiological studies show that the incidence or age specific prevalence of dementia has declined in the past 20 years [43–45], implying that the risk of dementia is modifiable through vascular risk reduction therapies. The early identification of patients at greatest risk of cognitive decline, by measuring change in cognition, is an important issue to be addressed by the PREVENT study.

**Fig. 1:**
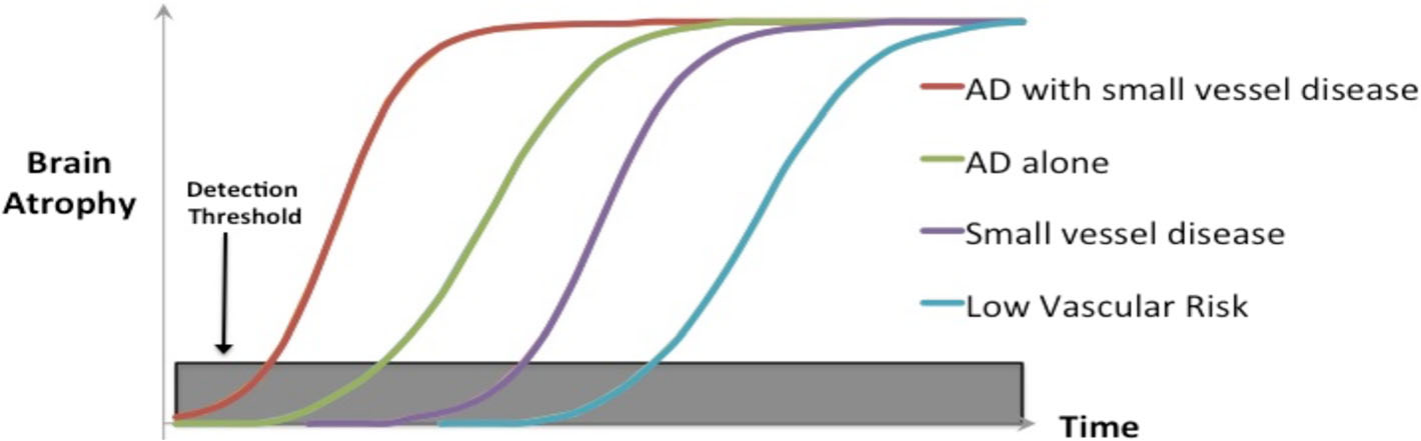
PREVENT stratification of dementia risk by biomarker profile for future dementia prevention clinical trials

The PREVENT study is expected to support the concept that rate of cerebral atrophy is a meaningful measure of disease progression that precedes cognitive decline and provide justification for the use of rates of cerebral atrophy as an outcome in pre-clinical dementia prevention trials. The PREVENT study in its design will stratify dementia risk according to biological markers of neurodegeneration, and small vessel cerebrovascular disease. We anticipate that the PREVENT study will demonstrate the temporal order of biomarker (in vivo AD and small vessel disease features) change as shown in the figure below with more advanced decline of brain volume occurring in order as follows: Alzheimer’s pathology plus cerebrovascular disease, Alzheimer’s disease in isolation, small vessel cerebrovascular disease and low vascular risk group without identifiable in vivo markers of either vascular disease or AD, all starting before clinical symptoms manifest (Fig. 1).

We anticipate that recruitment will be the most important challenge to the feasibility of the PREVENT Study. Clinical evaluations, cognitive tests, and the MRI protocol have been designed to minimize patient fatigue, and will be only performed yearly (inclusive of year 5). Recruitment is ahead of schedule providing powerful evidence that enrollment of TIA patients and healthy controls will be complete by the end of 2018. We are actively recruiting from a repository of healthy community volunteers (Calgary Normative Study). We anticipate that by the end of 2019 we will have completed our primary outcome measure of early rates of brain atrophy (R1). By 2021, secondary outcome measures of rates of brain atrophy will have been completed 3 years (R2) and biomarkers of Alzheimer’s disease in WML will have been analyzed. All patients will have completed 4 years of serial neuropsychological assessments.

Our sample size calculations for the PREVENT study are inclusive of an estimated attrition rate of 20%, which is similar to drop out rates in other longitudinal studies of cerebrovascular disease and cognition [46]. Potential bias of our results through the withdrawal or death of subjects at highest risk of dementia will be mitigated by the age inclusion criteria (45–75). Moreover, unlike stroke patients, TIA patients will be able to physically and cognitively comply with serial cognitive and MR imaging. All study participants will be under regular surveillance during clinical reassessments for the development of cardiovascular events and death. Confirmed cases of recurrent stroke will be excluded from our primary outcome analysis; recurrence is anticipated to be low (< 2%) [47].

A proportion of PREVENT subjects may have diffusion-weighted lesions (possibly up to 30%) [14], but previous studies have shown that presence of such small silent infarcts may not explain the cognitive profile [48], nor the progressive cognitive decline [49], perhaps implicating neurodegenerative and inflammatory processes [50, 51]. The presence of DWI lesions may be another predictor of progressive brain atrophy. Exploring disease mechanisms implicated in the recently reported interactions between incident stroke and persistent progressive cognitive decline will be important to furthering our understanding of the disease interaction of neurodegenerative and cerebrovascular processes on cognitive decline [49].

We have set rigorous standards for maintaining the highest quality MR images, including the application of similar benchmark standards used by the Alzheimer’s Disease Neuroimaging group (adni.loni.usc.edu). Objective quality assurance to detect image distortion will be diligently followed to measure properties that include grading calibration, image contrast, and signal to noise ratio. Any scans that fail quality control will be repeated within 4 weeks.

The PREVENT study will be the first critical step for identifying individuals at increased risk of late life cognitive decline. Future steps will target individuals at the highest risk of dementia before symptoms develop, thereby identifying an enriched disease group to test preventative and disease modifying strategies in clinical trials that aim to reduce the microscopic brain tissue loss over a relatively short period of time, thereby optimizing the opportunity for preventing dementia in the future.

## Abbreviations

AD: Alzheimer’s disease
APOE: Apolipoprotein E
BP: Blood pressure
CSF: Cerebrospinal fluid
DWI: Diffusion weighted imaging
FINGER: Finnish Geriatric Interventions to Prevent Cognitive Impairment and Disability study
MRI: Magnetic resonance imaging
MS: Multiple sclerosis
PREVENT: Predementia Neuroimaging of Transient Ischemic Attack study
QSM: Quantitative susceptibility mapping
TIA: Transient ischemic attack
VaD: Vascular Dementia
VCI: Vascular cognitive impairment
WHO/UCLA AVLT: World Health Organization/University of California Los Angeles auditory verbal learning test
WML: White matter lesion
